# Challenges and Solutions in Low-Biomass Respiratory Microbiome Profiling: A Workflow for Bronchoalveolar Lavage Fluid Sequencing in Guinea Pigs

**DOI:** 10.33549/physiolres.935728

**Published:** 2026-04-01

**Authors:** Tatiana BURJANIVOVA, Tomas BUDAY, Daniela MOKRA, Zuzana HANZLIKOVA, Eva BABUSIKOVA, Petar PODLESNIY, Veronika HOLUBEKOVA, Jaroslav BUDIS, Tomas SZEMES, Jana PLEVKOVA

**Affiliations:** 1 Department of Molecular Biology and Genomics, Jessenius Faculty of Medicine in Martin, Comenius University Bratislava, Martin, Slovakia; 2 Department of Pathophysiology, Jessenius Faculty of Medicine in Martin, Comenius University Bratislava, Martin, Slovakia; 3 Department of Physiology, Jessenius Faculty of Medicine in Martin, Comenius University Bratislava, Martin, Slovakia; 4 Geneton Ltd., Bratislava, Slovakia; 5 Department of Molecular Biology, Faculty of Natural Sciences, Comenius University Bratislava, Bratislava, Slovakia; 6 Institute of Clinical and Translational Research, Biomedical Research Center, Slovak Academy of Sciences, Bratislava, Slovakia; 7 Department of Medical Biochemistry, Jessenius Faculty of Medicine in Martin, Comenius University Bratislava, Martin, Slovakia; 8 Instituto de Investigaciones Biomédicas de Barcelona (IIBB), CSIC, Barcelona, Spain; 9 Biomedical Centre Martin, Jessenius Faculty of Medicine in Martin, Comenius University Bratislava, Martin, Slovakia; 10 Slovak Center of Scientific and Technical Information, Slovakia; 11 Science Park, Comenius University Bratislava, Bratislava, Slovakia; 12 Center for Medical Education Support, Jessenius Faculty of Medicine in Martin, Comenius University Bratislava, Martin, Slovakia

**Keywords:** Respiratory microbiome, Next-generation sequencing, 16S rRNA, Guinea pig, Low-biomass samples

## Abstract

Accurate profiling of the respiratory microbiome in low-biomass samples remains technically challenging due to host DNA contamination and limited microbial yield. This study aimed to optimize a methodological workflow for 16S rRNA sequencing of bronchoalveolar lavage fluid (BALF) obtained from guinea pig models with differing microbial statuses—specific pathogen-free (SPF) and conventionally bred (CON) animals. Using a comparative approach, we evaluated six commercial DNA extraction kits and tested different input DNA concentrations (1 ng vs. 0.5 ng) to enhance microbial detection while minimizing host DNA interference. Among the tested kits, only the ZymoBIOMICS DNA Microprep Kit yielded sufficient microbial DNA for downstream analysis. Real-time PCR and droplet digital PCR confirmed the microbial origin of the extracted DNA. Sequencing libraries were prepared from the V1–V3 regions of 16S rRNA genes and sequenced using the Illumina iSeq 100 platform. Taxonomic assignment and diversity metrics were analyzed using the MicrobAT pipeline. Our findings revealed significant differences in microbial composition between SPF and CON animals, notably in Mycoplasma abundance, which dominated the microbiota in CON but was nearly absent in SPF animals. Alpha and beta diversity metrics showed consistent stratification by animal group and input DNA concentration. However, a high proportion of unclassified reads—particularly in SPF samples—correlated strongly with sequences mapping to the Cavia porcellus genome, indicating substantial host DNA contamination. This study demonstrates the feasibility of microbiome profiling from low-biomass BALF samples in guinea pigs while highlighting the limitations of current sequencing and bioinformatic tools in distinguishing microbial from host-derived DNA. Our optimized workflow supports future respiratory microbiome studies in animal models and provides a foundation for improving host DNA depletion and reference databases tailored to non-human species.

## Introduction

Microbial colonization in multicellular organisms, shaped over millions of years of co-evolution, includes bacteria, viruses, yeasts, protozoa, and archaea. These microorganisms predominantly colonize mucosal and epithelial surfaces, forming diverse communities collectively referred to as the microbiota. The microbiota exhibits both long-term stability and the capacity for rapid fluctuation in response to environmental, dietary, immunological, and therapeutic factors [1]. While individual microbiomes can remain relatively stable over time, they are also dynamic and highly responsive to internal and external stimuli.

The concept of a “normal” or “healthy” microbiome has been increasingly challenged. Initial efforts to define a universal “core” microbiome—comprising microbial taxa shared across all individuals—have been met with significant variability in microbial composition between individuals [2] and within the same individual over time. This variability undermines the notion of a universal core, suggesting that microbial functionality, rather than taxonomic composition, may be more relevant for host physiology [3].Historically, the lower respiratory tract, including the lungs, was considered sterile. However, advances in molecular biology techniques, particularly next-generation sequencing, have revealed the presence of diverse microbial communities even in the lower airways. This paradigm shift occurred in 2010, with a study demonstrating the presence of microorganisms in the lungs of both healthy individuals and patients with bronchial asthma and chronic obstructive pulmonary disease (COPD) [4]. Based on this finding, as well as many more [5–7], the respiratory microbiota appears to play a significant role in maintaining homeostasis, interacting with host defense and/or protection mechanisms such as mucociliary clearance, cough, and both innate and adaptive immunity. Dysbiosis or disruption of these interactions may contribute to the pathogenesis of airway diseases [8,9], which are extensively studied in various animal models, e.g., acute lung injury, to elucidate further the complex pathogenesis of respiratory pathologies [10,11].

Animal models, especially rodents, are widely used in preclinical respiratory research due to their genetic manipulability, well-characterized immune systems, and physiological similarities to humans. These models are instrumental in investigating the pathophysiology of diseases such as chronic cough [12], asthma, COPD, and pulmonary fibrosis. They facilitate the exploration of molecular and cellular mechanisms underlying respiratory conditions, including immune responses and gene–environment interactions, through approaches such as targeted drug delivery and genetic modification.

In our previous studies using guinea pig models, we confirmed a relationship between microbial status and airway defense reflexes, such as cough [13], as well as breathing pattern regulation and cytokine production [14]. These findings were observed in both conventionally bred and specific pathogen-free (SPF) animals – animals that are bred and maintained in controlled environments to ensure that they are free from particular pathogens that could interfere with research outcomes [15]. We hypothesized that differences in airway microbiota diversity may be a critical factor underlying these differences [13,14].

The present study aims to develop an optimized protocol for 16S rRNA sequencing of airway microbiota from bronchoalveolar lavage fluid (BALF) obtained from two guinea pig models with vendor-defined differences in microbial status. This methodological development seeks to facilitate omics-based analysis of the airway microbiome, enabling deeper insights into host–microbe interactions and informing the development of novel preclinical models that leverage microbiota manipulation.

## Materials and Methods

### Ethical policy statement

The study was approved by the Ethics Committee of the Jessenius Faculty of Medicine in Martin (EK 65/2022), Comenius University, Bratislava, Slovakia, and was conducted in accordance with the institutional guidelines on animal care and the laws and legislation of Slovakia. The approved experimental protocol complies with the EU Directive 2010/63/EU on animal experiments.

### Laboratory animals

Male and female Dunkin Hartley guinea pigs were used (n=7; age 6–7 weeks; Animal Breeding Facility JFM CU, Slovakia) – conventionally bred animals (CON) with the intact microbiome, as well as specific pathogen-free (SPF) animals (n=9; age 6–7 weeks; Charles River Laboratories, France). The confirmation of the SPF status was provided by Research Animal Diagnostic Services Europe, Lyon, France (primary laboratory for examination, necropsy, and direct and culture) and Research Animal Diagnostic Services, Wilmington, United States (primary laboratory for MFIA and PCR). The animals were housed in accredited animal facilities with standard rodent enrichment recommended by animal welfare bodies, an ambient temperature of 20–24 °C, and humidity control of 45–65 %, with an alternating 12-hour light/dark cycle. They received standard food and water enriched with vitamin C ad libitum.

### Biological material sampling post-mortem

The animals were euthanized by CO_2_ asphyxiation with subsequent exsanguination by a direct puncture of the heart.

The lungs were lavaged twice through the bronchus with sterile saline (10 ml/kg b.w.). The recovered lavage fluid was centrifuged at 1500 g for 15 min at 4 °C. Supernatants from the lavage fluid were frozen and stored at −80 °C prior to analysis.

### DNA extraction

Several verified procedures in the literature exist for obtaining and characterizing DNA from the microbiome; however, most of these procedures focus on the microbiome from the human gastrointestinal tract and stool. Six different microbial DNA isolation methods (QIAamp DNA Microbiome Kit, DNeasy PowerSoil Pro Kit, QIAamp DSP Virus Kit, MAGicBead™ cfDNA Isolation Kit (Qiagen), ZymoBIOMICS DNA Miniprep Kit, and ZymoBIOMICS DNA Microprep Kit for BALF samples were tested. Genomic DNA was isolated according to the manufacturer’s protocol for the kits listed above. The Qubit dsDNA HS assay kit (Life-Technologies, Carlsbad, California, USA) and the Qubit 2.0 fluorometer (Invitrogen, Waltham, Massachusetts, USA) were used for DNA quantification.

### Identification of the presence of bacterial DNA

#### Real-time PCR detection

The quantity of DNA was determined using the fluorometric method on the Qubit 4.0 (Invitrogen). The DNA was diluted, and 10 ng was used in real-time PCR analysis to detect the presence of bacterial DNA using an assay developed for the evolutionarily conserved region of the 16S rRNA gene (Microbial DNA qPCR Assay, Qiagen) according to the manufacturer’s protocol. The assay enables amplification, resulting in a positive test result for species within the seven ongoing phyla: *Actinobacteria, Bacteroidetes, Euryarchaeota, Firmicutes*, *Fusobacteria, Proteobacteria*, and *Tenericutes*. Real-time PCR was performed on a QuantStudio 5 instrument (Applied Biosystems) with thermal cycling that included an initial denaturation at 95 °C for 10 minutes, followed by 40 cycles of denaturation at 95 °C for 15 seconds, and annealing and extension at 60 °C for two minutes. All samples were analysed using the instrument software according to the manufacturer’s recommendations.

#### 16S rDNA droplet digital PCR detection

**Fig. 1:**
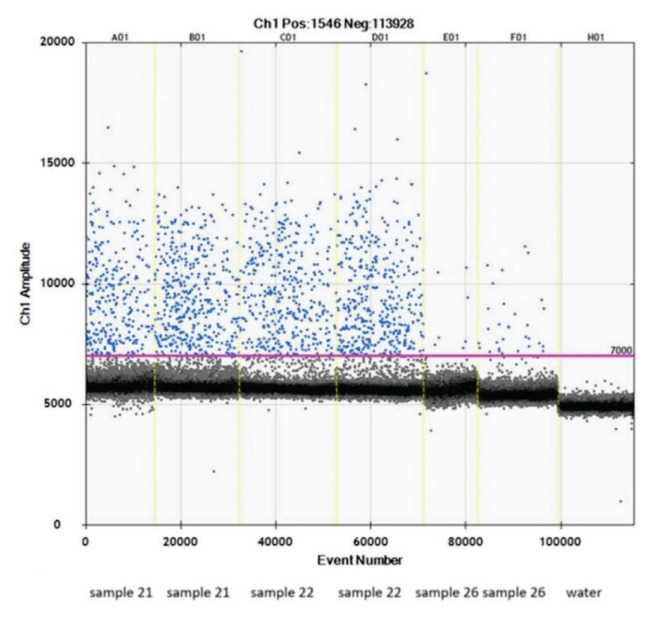
Demonstration of microbial DNA detection using ddPCR. Representative 1-D plot of ddPCR reactions for the V4 region of the 16S rRNA. Samples labelled 21, 22, and 26 were analysed using ddPCR in duplicate, with water serving as a negative control.

Twenty microliters of ddPCR (droplet digital PCR) reaction were prepared. This mixture contained 19 μl of mastermix, composed of 10 μl of EvaGreen Supermix, 2 μl of forward and reverse primers targeting the V4 region of the 16S rRNA (final concentration 50 nM), and 7 μl of ultrapure water (Fig. 1). Then, 1 μl of isolated DNA was added to the mastermix. The primers’ sequences used for this study were 16SF as a forward primer [5′-GTGYCAGCMGCCGCGGTAA -3′] and 16SR as a reverse primer [5′-GGAC TACNVGGGTWTCTAAT -3′]. Droplet digital PCR was performed in duplicate on each sample. In each run, a negative control, 1 μl of ultrapure water, was run with each mastermix to exclude contamination of the reagents. Final volume (20 μl) was pipetted into the middle rows of the DG8™ Cartridge (Bio-Rad Laboratories, Hercules, California, USA). Then, 70 μl of Droplet Generation Oil for EvaGreen was applied to the bottom wells. The DG8™ Cartridge was then placed in the QX200™ Droplet Generator (Bio-Rad Laboratories, Hercules, California, USA), and 40 μl of generated droplets were pipetted from the upper wells into the 96-well plate. The PCR plate was covered with a pierceable foil and heat-sealed using Bio-Rad PX1™ (Bio-Rad Laboratories, Hercules, California, USA). The PCR plate was then placed in a T100™ thermal cycler (Bio-Rad Laboratories, Hercules, California, USA). Droplets were generated by an automated droplet generator (Bio-Rad Laboratories, Hercules, California, USA) according to the manufacturer’s instructions. PCR was performed using a T100 thermal cycler (Bio-Rad Laboratories, Hercules, California, USA) with the following cycling conditions: denaturation at 95 °C for 5 min, followed by 40 cycles of denaturation at 95 °C for 30 s, followed by annealing at 55 °C for 30 s, extension at 72 °C for 120 s, cooling at 4 °C for 5 min and droplet stabilization at 90 °C for 5 min. Samples were then analyzed using QX200 Droplet Reader (Bio-Rad Laboratories, Hercules, California, USA). Thresholding was carried out by using QuantaSoft Software manually at the lowest amplitude that captures true negative clusters based on the signals of the negative control and positive control samples.

#### Library preparation and 16S rDNA sequencing

The V1–V2–V3 regions of the microbial 16S rDNA genes were amplified using Microbiota solution A (Arrow Diagnostics, Genova, Italy) according to the manufacturer’s instructions. The library *quality* was assessed using the Agilent Bioanalyzer 2100 DNA High Sensitivity DNA kit (*Agilent*), and the concentration was tested with Qubit^®^ dsDNA HS (High Sensitivity) assay kit (Invitrogen Co., Life Sciences, Carlsbad, USA). These steps serve as a preparation for the pooling procedure consisting of equimolar libraries. The final loading concentration of the library was 120 pM. The sequencing libraries were initially prepared according to the manufacturer’s recommendations using an input DNA concentration of 1 ng. Due to the high proportion of host DNA in some samples, we optimized the protocol for a subset of SPF (Specific Pathogen Free) animals by reducing the input DNA concentration to 0.5 ng. The aim of this modification was to reduce the amount of host DNA in the sequencing libraries, thereby increasing the coverage of the microbial genome. It was done to evaluate the impact of the reduced amount of input material on the quality of the resulting libraries. Sequencing of our libraries was performed on an iSeq 100 Illumina^®^ sequencing platform (Illumina, CA, US) using the V2 300 cycles reagent.

### Data processing and statistical analysis

Asymmetrically sequenced data (50 bp and 250 bp reads) were analyzed using the bioinformatics MicrobAT software (MicrobAT Suite – SmartSeq, Novara, Italy), which relies on data from the RDPdb database version 11.4, and its usage as an analytical tool for the Arrow pipeline is strongly recommended and standardized by many publications. The MicrobAT tool provided read counts assigned to various taxonomic levels, down to the species level or to higher-level unclassified taxa where species-level classification was not possible. To provide a more comprehensive view, we extracted additional metrics from the analysis tool to describe microbial alpha diversity in the samples. These included the Shannon index, equitability (evenness), Chao1 richness estimator, Gini-Simpson index, Simpson dominance index, and the reciprocal Simpson index.

To gain deeper insights into the origin of unclassified reads, we mapped the sequences to the reference genome of *Cavia porcellus,* version mCavPor4.1 [16], based on the hypothesis that these unclassified reads may originate from the host itself and represent sample contamination by host DNA.

## Results

### Microbial DNA isolation and detection results

Of the six isolation kits tested, which we described in the methods section, only the ZymoBIOMICS DNA Microprep Kit provided a measurable DNA yield after the isolation. Real-time PCR and digital droplet PCR (ddPCR) methods were then used to verify the microbial origin of the isolated DNA. The isolated microbial DNA was detected by both Real-time PCR and the more sensitive ddPCR (Fig. 1). Both input concentrations (0.5 ng and 1 ng) were suitable for further bioinformatic analysis.

### Composition of the microbiome

Among the specific pathogens at the genus level: *Pasteurella, Salmonella, Clostridium*, *Helicobacter*, and *Mycoplasma*, which are typically used to differentiate SPF from conventionally-bred *Cavia porcellus*, a notable effect was observed only for *Mycoplasma* (including *Candidatus Mycoplasma ravipulmonis*, *Mycoplasma molare (T)*, *Mycoplasma mobile 163K*, and unclassified *Mycoplasma*) [17]. In conventional animals, *Mycoplasma* accounted for an average of 96.62 % of classified reads (i.e., all reads entering classification minus unclassified Bacteria at the phylum level) and 31.34 % of all reads entering classification. In contrast, SPF animals showed only 2.08 % of classified reads and 0.02 % of total reads attributed to Mycoplasma for 1 ng concentration and 3.28 %, respectively 0.06 % for 0.5 ng concentration. The other listed genera were not detected in either conventional or SPF animals, except for a single SPF sample (0.5 ng concentration) in which *Clostridium* was detected at 0.02 % of classified reads, likely representing an outlier observation.

**Fig. 2:**
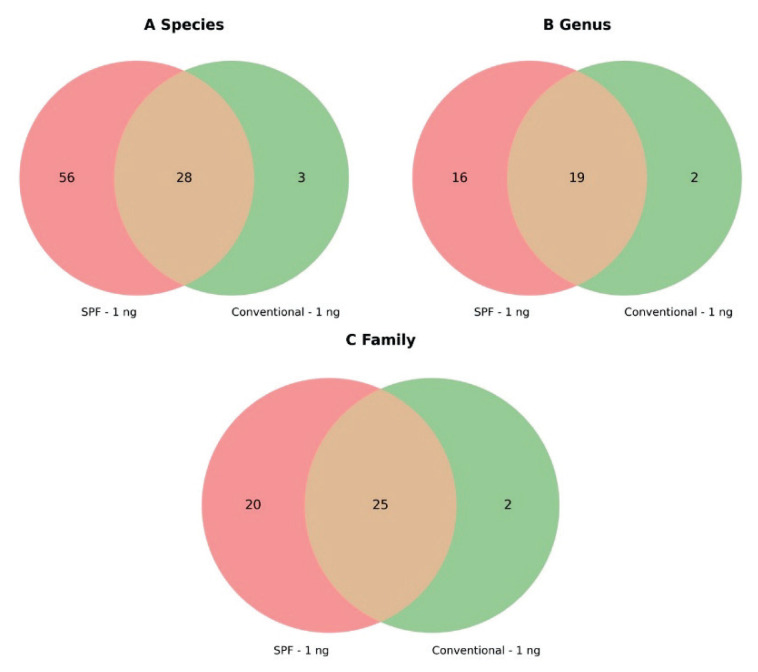
Overlap of unique taxonomic classifications present in more than one sample between SPF and conventional samples after using ZymoBIOMICS DNA Microprep Kit with input concentration 1 ng: **A**) Species level; **B**) Genus level; **C**) Family level

A total of 425 unique species-level taxonomic classifications were identified across all samples, of which 331 remained after excluding unclassified species assigned only to higher taxonomic ranks. Among these, only 122 species were present in more than one sample, regardless of group assignment (Fig. 2A).

At the genus level, 169 unique classifications were observed, reduced to 97 after excluding unclassified genera. Of these, only 44 were detected in more than one sample across groups (Fig. 2B).

At the family level, 96 unique classifications were identified, 72 of which remained after excluding unclassified entries. Only 51 families were shared by more than one sample, independent of sample grouping (Fig. 2C).

**Fig. 3:**
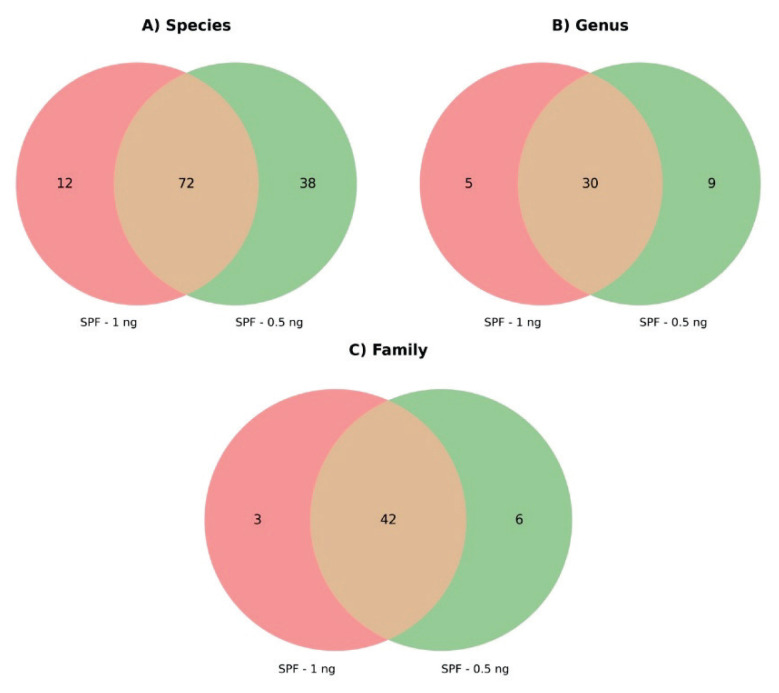
Overlap of unique taxonomic classifications present in more than one sample between SPF samples after using ZymoBIOMICS DNA Microprep Kit with input concentration 0.5 and 1 ng: **A**) Species level; **B**) Genus level; **C**) Family level

The distribution of individual taxa across samples, reflected in biodiversity indices, indicates that the BALF of SPF animals serves as a source of microbial taxa not typically found in conventional animals. Notably, using a lower input DNA concentration of 0.5 ng further increased species richness (Fig. 3).

### Microbiome diversity

#### Alpha diversity

**Fig. 4:**
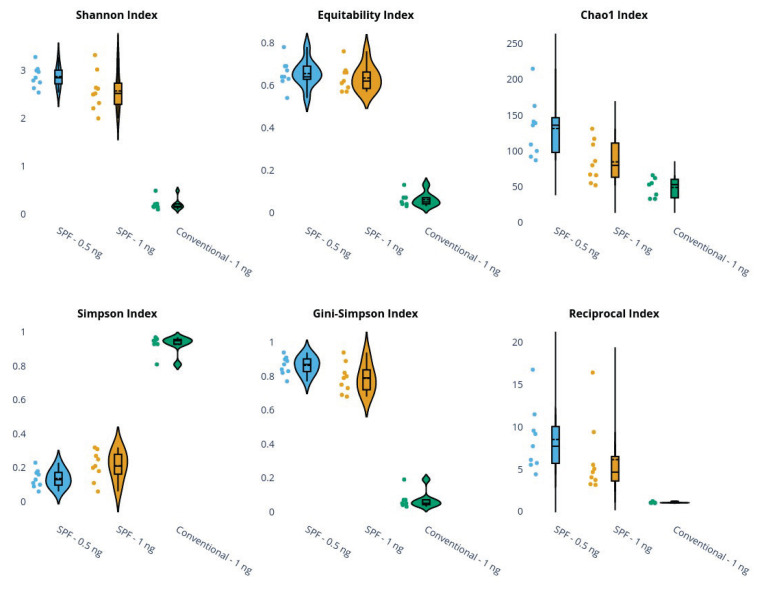
Violin plots of alpha diversity metrics

We examined the variability of alpha diversity metrics across sample subgroups (Fig. 4). Statistical testing of the hypothesis of equality between SPF and conventional animals (DNA samples for library preparation were diluted to a starting concentration of 1 ng) means using the Mann–Whitney U test (α = 0.05) revealed significant differences in all biodiversity metrics between SPF and conventional animals.

For the paired comparison of SPF samples extracted using concentrations 0.5 and 1 ng, we first assessed data normality and subsequently applied either a paired t-test or the Wilcoxon signed-rank test, as appropriate. All tested diversity indices, except for the Equitability index, showed statistically significant differences.

However, these results appear to be strongly influenced by considerable variation in total read counts, both within and between groups, with SPF animals yielding substantially higher sequencing depths. Although diversity indices are designed to partially account for variation in sequencing depth, increasing total read counts inherently raises the statistical and biological likelihood of detecting higher diversity. For example, the correlation between total read count and the Gini–Simpson index was 0.70, highlighting the potential confounding effect of sequencing depth on diversity estimates.

#### Beta diversity

Applying rarefaction in this context did not appear to be appropriate, due to the high abundance of incomplete or unclassified reads and generally low read depth across samples. Rarefaction would have resulted in substantial data loss and distortions when down-sampling to match the sample with the lowest number of reads. For this reason, we opted for an alternative approach to evaluate beta diversity - Principal Coordinates Analysis (PCoA) based on Bray-Curtis dissimilarity matrices computed from relative abundance data. This analysis excluded unclassified categories but preserved their proportional contribution to the total read count. The procedure was applied independently for taxonomic levels Species, Genus, and Family.

**Fig. 5:**
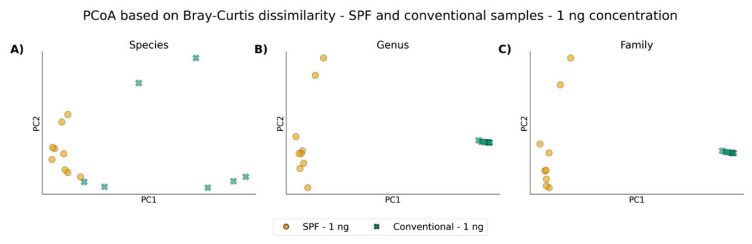
PCoA based on Bray-Curtis dissimilarity matrices between SPF and conventional samples after using ZymoBIOMICS DNA Microprep Kit with input concentration 1 ng: **A**) Species level; **B**) Genus level; **C**) Family level

**Fig. 6:**
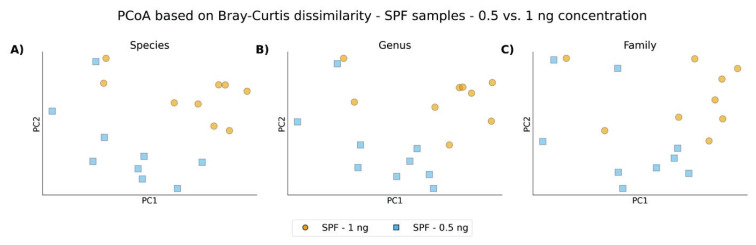
PCoA based on Bray-Curtis dissimilarity matrices between SPF samples after using ZymoBIOMICS DNA Microprep Kit with input concentration 0.5 and 1 ng: **A**) Species level; **B**) Genus level; **C**) Family level

Across all taxonomic levels, the ordination plots reveal an almost clear clustering pattern distinguishing conventional and SPF animals, regardless of the taxonomic resolution. Additionally, a partial clustering of SPF samples can be observed, which appears to be influenced by the amount of input DNA during library preparation (Fig. 5, 6).

**Fig. 7:**
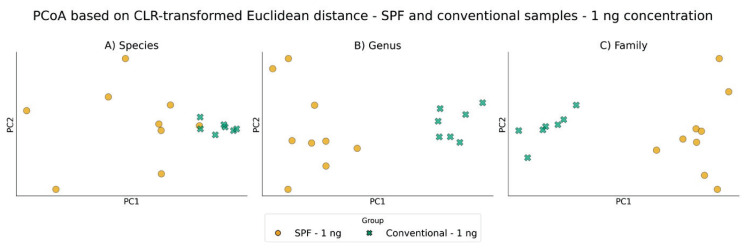
PCoA based on CLR-transformed Euclidean distance between SPF and conventional samples after using ZymoBIOMICS DNA Microprep Kit with input concentration 1 ng: **A**) Species level; **B**) Genus level; **C**) Family level

**Fig. 8:**
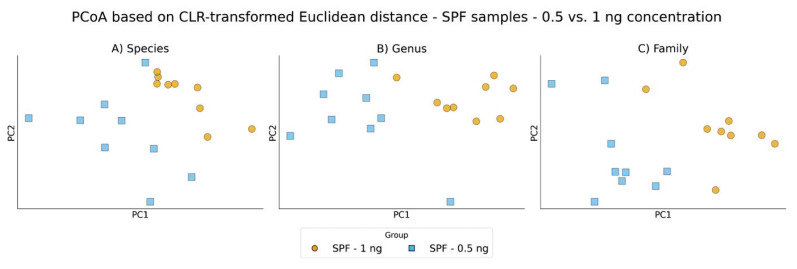
PCoA based on CLR-transformed Euclidean distance between SPF samples after using ZymoBIOMICS DNA Microprep Kit with input concentration 0.5 and 1 ng: **A**) Species level; **B**) Genus level; **C**) Family level

Comparable clear clustering patterns and group separations were observed when using Euclidean distances computed after centered log-ratio (CLR) transformation of the same relative abundance data, supporting the robustness of the observed trends (Fig. 7, 8).

### Unclassified reads issue

Overall, the interpretation of these results is not straightforward. Taxonomic counts generated by the MicrobAT tool included a substantial proportion of (partially) unclassified reads at various taxonomic levels. The internal biodiversity metrics were computed by the tool after excluding unclassified bacteria at the Phylum level, which, on average, represented 98.75 % of reads assigned to taxa per sample in SPF animals at a concentration of 1 ng, respectively 97.75 % at a concentration of 0.5 ng, and 67.79 % in conventional animals. Moreover, the samples contained reads that were not classified down to the Species level, making it unclear how many distinct organisms—ranging from 1 to n, where n is the number of unclassified reads—are included within such broadly defined categories. It is also uncertain whether these reads represent novel, unique taxa or belong to existing classified taxa. Nonetheless, the tool treats these unclassified categories as single homogeneous groups, which may lead to an underestimation of actual biodiversity by ignoring the potential variation within these groups.

In addition, the number of reads mapped to *Cavia porcellus* genome showed a strong correlation with the number of “Unclassified Bacteria and Archae” reads (Pearson r = 0.97), especially those with high mapping quality (MAPQ ≥ 30), which correlated negatively (r = −0.89).

These results may be interpreted in two ways: (1) the unclassified reads represent host DNA contamination from *Cavia porcellus*, and/or (2) the sequences—particularly those related to 16S rRNA—are highly conserved between the host and certain microbial taxa, limiting the ability to accurately distinguish between host and microbial DNA.

## Discussion

This study provides a detailed characterization of the bronchoalveolar microbiome in guinea pigs using next-generation sequencing (NGS) of 16S rRNA amplicons, with a focus on methodological optimization for low-biomass respiratory samples. Our findings underscore both the potential and the limitations of current microbiome profiling techniques in the context of the lower respiratory tract. Comprehensive DNA/RNA testing with bioinformatic analysis of the results widely contributes to the understanding of pathogenesis of e.g. cardiac diseases or cancer alongside the respiratory diseases [18–20].

The lung microbiome presents with unique analytical challenges due to its inherently low microbial biomass and high host DNA content. These factors can significantly compromise sequencing depth, taxonomic resolution, and the reliability of diversity estimates [21, 22]. In the respiratory microbiome, DNA of host origin is highly likely to predominate, as our results have also shown. The presence of a high proportion of host DNA greatly complicates these analyses and poses a challenge not only for DNA collection but also for the interpretation of the data obtained [23].

In our study, host DNA contamination was a major confounding factor, particularly in SPF animals, where unclassified reads dominated the dataset. This observation aligns with previous reports indicating that host DNA can constitute over 90 % of total DNA in BALF samples [24]**.** A high proportion of unclassified reads in samples from SPF guinea pigs can be attributed to several interrelated factors: 1) SPF animals are bred to minimize microbial colonization, resulting in extremely low microbial biomass and a predominance of host DNA, which can obscure microbial signals and lead to ambiguous taxonomic assignments; 2) Current reference databases are often incomplete or biased toward human-associated microbes, limiting the ability to classify sequences from guinea pig-specific or rare taxa; 3) Low input DNA concentrations and high host DNA content can produce short or low-quality reads that fail to align confidently with known microbial sequences and simultaneously, the conserved nature of certain 16S rRNA regions may hinder resolution at the genus or species level [25]. The high proportion of unclassified reads—strongly correlated with reads mapping to the *Cavia porcellus* genome—suggests that host DNA depletion remains a critical unmet need in respiratory microbiome research, particularly using various bioinformatic approaches.

The MicrobAT pipeline used in this study provided robust taxonomic summaries but lacked per-read resolution and support for operational taxonomic unit (OTU) clustering. This limitation, combined with the high proportion of unclassified reads, likely led to an underestimation of true microbial diversity. Moreover, the inability to resolve ambiguous sequences may obscure the detection of novel or rare taxa, a known limitation of 16S rRNA-based approaches [26]. OTU grouping based on sequence similarity thresholds could mitigate the impact of sequencing artefacts (e.g., primer/adaptor bias or amplification errors) that may affect classification accuracy, particularly at lower taxonomic levels. Additionally, even 100 % identical 16S rRNA sequences can originate from different genomes, as discriminatory variation might lie outside the sequenced regions. The presence of unclassified reads may also reflect the limited representation of guinea pig-associated microbes in current reference databases. Expanding these databases with host-specific microbial genomes could improve classification accuracy and facilitate cross-species comparisons [27].

Our results revealed significant differences in alpha and beta diversity between SPF and conventionally bred guinea pigs. Notably, Mycoplasma spp. were highly abundant in conventional animals but nearly absent in SPF animals, consistent with their exclusion from SPF breeding environments [28]. Interestingly, reducing the input DNA concentration from 1 ng to 0.5 ng in SPF samples led to an increase in observed species richness. While this may reflect reduced host DNA interference, it also raises concerns about stochastic amplification effects and the influence of sequencing depth on diversity metrics [29].

The reduced resolution observed in metagenomic analysis of BALF samples is a consequence of technical limitations associated with low microbial biomass and high host DNA contamination. This ratio significantly reduces the sequencing depth for microbial sequences, limiting the detection of low-abundance taxa and reducing the overall phylogenetic and functional resolution of the analysis. Compared to gastrointestinal tract samples, where the microbial fraction is highly dominant and diversity is naturally higher, BALF samples are technically more challenging to process and interpret and are also more susceptible to contamination from reagents and the environment.

The main sources of contamination are PCR reagents, molecular biology-grade water, and extraction kits. Detected contaminants often originate from environmental bacteria, particularly those associated with water and soil [21]. To minimize this contamination, the laboratory premises were regularly decontaminated, work surfaces were cleaned with DNA-degrading solutions, and isolations were performed in a laminar box equipped with UV sterilization. The workplaces were also regularly irradiated with UV light as part of a preventive regime [22].

For ddPCR, we utilized a PCR assay designed by us, featuring specific primers and probes, which were aimed at detecting defined microbial targets without amplifying host DNA. In both cases, the designed primers were validated for specificity and sensitivity. Molecular pure water was used as a negative control in all reactions to ensure monitoring of potential contamination of reagents or the laboratory environment.

## Limitations

In this study, the presence of microbial DNA was confirmed using our validated digital PCR assay, which enables sensitive detection of microbial targets even in low-biomass samples. We applied this assay to all analyzed samples, verifying that the sequenced samples contained detectable microbial DNA and were not exclusively host material. We consider this validation step necessary to minimize technical artifacts and increase the reliability of subsequent analyses, especially in standard microbial environments with high biomass, where the microbial signal is dominant. We plan to extend the methodological comparison to include alternative library preparation kits optimized for samples with low levels of microbial DNA. The aim of this future direction is to methodologically evaluate the differences among available approaches and better understand their impact on the quality of sequencing data and subsequent bioinformatic processing. These plans do not assume automatic improvement in results but reflect the need for further methodological optimization and standardization in lung microbiome research.

Moreover, the use of Arrow’s Microbiota Solution A in practice often entails the subsequent application of the MicrobAT tool for taxonomic classification of individual sequencing reads. However, several alternative tools exist (e.g., Kraken, QIIME 2) that rely on different reference databases and apply distinct computational approaches to the technical classification of reads. For example, Kraken2 performs k-mer–based classification using user-defined reference databases, which are commonly built from NCBI RefSeq or GenBank genomes, whereas QIIME 2 typically assigns taxonomy based on curated marker-gene databases such as SILVA, Greengenes, or GTDB, depending on the chosen classifier and configuration. Consequently, if it were possible to avoid the Arrow workflow already at the library preparation stage, a broader range of downstream bioinformatic tools and databases could be employed. This would increase methodological flexibility and allow for more comprehensive comparisons across analytical pipelines, potentially improving the robustness and interpretability of the results. Such an approach might also facilitate the taxonomic assignment of reads that currently remain unclassified, at least at higher taxonomic levels.

## Conclusions

The presented methodology for omics-based microbiome assessment in BALF from animal models has been demonstrated to identify the differences between the distinct groups of animals by optimizing DNA input concentrations and comparing different extraction kits, as evidenced by multiple diversity indices.

Our results emphasize the necessity of validated methodologies in low-load microbiome research and provide valuable insights into the microbial ecology of the respiratory tract in animal models.

This methodology can be used to track changes in microbiome diversity in the further development of animal models utilizing microbiota manipulation (e.g., antibiotic-induced or allergen-induced dysbiosis, the effect of probiotic supplementation, or bacteriophage therapy) and the observation of these effects on the respiratory system as a whole.

## Data Availability

The datasets generated and/or analyzed during the current study are available in the NCBI BioProject repository, https://www.ncbi.nlm.nih.gov/bioproject/PRJNA1333852.
